# Is the End-Exercise Potentiated-Twitch Decrement a Regulated Variable? A Mechanism-Oriented Review of Muscle Fatigue During High-Intensity Aerobic Exercise

**DOI:** 10.3390/muscles5040074

**Published:** 2026-10-09

**Authors:** Arnulfo Ramos-Jiménez, Mariazel Rubio-Valles, Marina Trejo-Trejo, Cristhian Emmanuel López-Campos, Jaime Güereca-Arvizuo, Oliver Adán Sollano-Trejo, María Guadalupe Delgadillo-Ramos, Marco Antonio Hernández Lepe

**Affiliations:** 1 Departamento de Ciencias de la Salud, Instituto de Ciencias Biomédicas, Universidad Autónoma de Ciudad Juárez, Ciudad Juárez 32401, Chihuahua, Mexico; aramos@uacj.mx (A.R.-J.); jaime.guereca@uacj.mx (J.G.-A.); 2 Conahcyt National Laboratory of Body Composition and Energetic Metabolism (LaNCoCoME), Tijuana 22390, Baja California, Mexico; lupitadelgadillo@uabc.edu.mx; 3 Facultad de Ciencias de la Cultura Física, Universidad Autónoma de Chihuahua, Chihuahua 31125, Chihuahua, Mexico; p305510@uach.mx; 4 Facultad de Deportes Campus Mexicali, Universidad Autónoma de Baja California, Mexicali 21289, Baja California, Mexico; marina.trejo@uabc.edu.mx (M.T.-T.); lopez.cristhian92@uabc.edu.mx (C.E.L.-C.); oliver.sollano@uabc.edu.mx (O.A.S.-T.); 5 Facultad de Medicina y Psicología, Universidad Autónoma de Baja California, Tijuana 22390, Baja California, Mexico

**Keywords:** muscle fatigue, physical endurance, athletic performance, afferent pathways, sarcoplasmic reticulum

## Abstract

Background: Fatigue in high-intensity exercise is often attributed to a defended ceiling of peripheral fatigue; evidence is mixed. Objectives: We aimed to assess whether the end-exercise potentiated-twitch decrement behaves as a regulated variable across candidate mechanisms. Methods: PubMed/MEDLINE, Scopus, and Web of Science were searched to 12 August 2026. Evidence came from controlled studies in adults, with permeabilized-fiber, rodent, and isolated-actomyosin experiments only where human measurement is impossible. Results: Nine mechanisms met the criteria. Inorganic phosphate and hydrogen ions impair the cross-bridge cycle; reduced sarcolemmal excitability (K^+^) and reduced sarcoplasmic reticulum Ca^2+^ release reverse over minutes and days, respectively. Only the cross-bridge effects are driven by metabolites (protons, ATP, lactate) that are agonists of group III/IV afferents; the afferents do not sense contractile impairment itself. Oxygen delivery, respiratory-muscle work, substrate availability, and perceptual load altered fatigue rate more than the end-exercise value, which varied with duration, prior exercise, muscle mass, and mode. Conclusions: Evidence fits a threshold in the phosphate–force relationship, though this does not demonstrate a defended limit. The decrement is not constant across tasks; we propose, as a testable hypothesis, that the regulated quantity is the summed afferent and effort signal. Selection was purposive, without formal risk-of-bias appraisal.

## 1. Introduction

Fatigue determines the outcome of endurance competition, and assumptions about its cause shape how athletes are prepared. If exercise ends because contractile function has fallen to a fixed limit that the nervous system defends, training and nutrition should aim to raise that limit; if it ends because an integrated sensory signal has become intolerable, they should instead slow the accumulation of that signal. Set out this way, the two accounts look mutually exclusive, and they are not. A single metabolite can occupy both roles at once. Inorganic phosphate (Pi) depresses contractile function directly, and it has been proposed as an object of regulation: in rodent muscle, peak twitch force does not begin to fall until intramuscular Pi passes a threshold concentration [1], a breakpoint that shows a nonlinear concentration–force relationship but does not by itself demonstrate a defended limit, and Pi accumulates alongside the protons that are agonists of the group III/IV afferents restraining central motor output [2,3]. A system that defends an upper limit for intramyocellular Pi and a system in which Pi contributes to metabo-sensing intolerable to full neural activation are therefore complementary descriptions, not competing ones. What this review contests is narrower and concerns the measurement rather than the physiology: whether the potentiated-twitch decrement, the composite index used to quantify peripheral fatigue, can itself be the regulated variable. If it can, training and nutrition should aim to raise the limit it defines; if it is instead a sum of processes with shifting weights, only some of which arise from stimuli the afferents sense, then the target is the rate at which that sensed fraction accumulates, and the index will differ across tasks even when regulation is intact [4]. The question also governs how fatigue is measured, because almost every study operationalizes it as the post-exercise fall in potentiated quadriceps twitch force and treats that value as though it had a defined set point.

Two claims are at issue, and they are not equally supported. The first is that group III/IV muscle afferents reduce central motor output; the second, stronger, is that this feedback keeps peripheral fatigue near a fixed end-exercise value within a given task; its proponents do not claim a universal value across tasks. The evidence behind them is incomplete where it exists and contradictory where it has been tested directly. The same index moves in opposite directions under the same manipulation: as cycling time trials lengthen, the end-exercise twitch decrement falls (40%, 31%, and 29%) [4], whereas as trail races lengthen, it rises (−14 ± 10% to −28 ± 14%) [5]; halving the exercising muscle mass raises it [6]; and prior arm exercise lowers it while shortening tolerance [7]. What has never been measured matters as much: afferent traffic and contractile impairment have not been recorded simultaneously in exercising humans, so the regulatory link is inferred rather than observed, and no experiment has compared durations in trained athletes with the twitch obtained at cessation rather than after the 1 to 2 min in which it recovers at task-dependent rates [8,9].

The gap lies in what the second claim assumes about its own measurement. It treats the potentiated-twitch decrement as a single physiological quantity with a set point. It is not: it is the summed output of processes that differ in reversibility by three orders of magnitude, are driven by different stimuli, and are not equally sensed by the afferents that carry the feedback. Neither position in the debate questions the index itself. This review does. It examines each candidate mechanism and its evidence in turn, then asks what that evidence implies about fatigue regulation. It derives from decomposing the twitch index and identifies the measurements that would falsify the account proposed here.

Terms are used as follows. Muscle fatigue is the exercise-induced reduction in the force or power a muscle can produce, whether or not the task can be sustained. Peripheral fatigue is the fraction of that reduction arising at or distal to the neuromuscular junction, estimated here as the decrement in potentiated twitch force and referred to interchangeably as contractile impairment; central fatigue is the fraction arising proximal to the neuromuscular junction, estimated as the decrement in voluntary activation. Performance fatigability is the measured decline in an objective performance variable over the course of a task. Perceived effort is the conscious sensation of how hard the task is. Exercise tolerance is the time or work sustained before task failure, the point at which the required power or torque can no longer be produced. These are distinct outcomes; the argument below concerns whether the estimate of peripheral fatigue behaves as a regulated variable, not whether fatigue in any of these senses occurs.

## 2. Results: Candidate Mechanisms and the Evidence for Each

Nine candidate mechanisms follow. Each is assessed on four attributes, and the argument of this review depends on the fourth as much as on the first three: the size of the measured change during exercise of the relevant intensity; whether an experimental manipulation has established that the change is causal rather than merely concomitant; the time course over which it reverses, which ranges across three orders of magnitude from minutes to days; and whether the stimuli that produce it are known agonists of the group III/IV muscle afferents that carry the feedback. The first three attributes are the conventional content of a fatigue review. The fourth is what separates the mechanisms that can contribute to a regulated sensory signal from those that depress force without being reported centrally, and it is the attribute on which the two competing accounts of exercise termination are usually silent. Because the potentiated-twitch decrement sums both sets indiscriminately, the mechanisms are presented here in order of increasing reversibility time, and each subsection closes by stating that time course and, where it is known, whether its proximate stimuli are sensed by these afferents. Section 3 then asks what a sum with these properties can and cannot be taken to measure. Two caveats apply throughout. No study has simultaneously recorded afferent traffic and contractile impairment in exercising humans, so the fourth attribute is inferred from the known agonists of these afferents [2] rather than observed. No study has quantitatively partitioned the twitch decrement among the mechanisms; the relative weights described below are qualitative. In addition, the evidence spans tasks ranging from brief all-out sprints and single-joint isometric contractions to ultra-distance running and several hours of moderate-intensity exercise, as well as in vitro preparations; these are treated as sources of mechanistic information about distinct tasks, not as interchangeable fatigue tasks.

### 2.1. Inorganic Phosphate and Hydrogen Ions

In 254 permeabilized myofibers from the vastus lateralis of 12 men, pH 6.2 with 30 mM Pi reduced isometric force, slowed unloaded shortening velocity, and depressed peak power, with the two ions acting together rather than singly [10]. Their molecular actions are separable, and separating them matters, because isometric tension and contractile velocity are not limited by the same step of the cross-bridge cycle. Pi depresses isometric force principally at the force-generating transition itself: it rebinds strongly bound actomyosin, reverses the power stroke, and returns myosin toward a pre-power-stroke state, so that fewer cross-bridges occupy high-force configurations at any instant. Laser-trap measurements of Pi binding to fast skeletal actomyosin show that this binding is both concentration- and load-dependent, which accounts for the saturating and strain-sensitive character of the effect [11]. Acidosis depresses isometric force by a different route, and to a smaller degree than early low-temperature fiber work implied: at pH 6.5, the average force-generating capacity of a myosin mini-ensemble fell by 20%, attributable both to less force generated per actomyosin interaction and to a greater number of non-productive binding events in which myosin attaches without completing a power stroke [12]. Shortening velocity is governed by detachment kinetics instead, and here acidosis is the more consequential ion. A mechanochemical model constrained by laser-trap data indicates that low pH slows ADP release, thereby slowing myosin’s detachment from actin, increasing non-productive rebinding of detached heads, and slowing the weak-to-strong binding transition by which heads attach [13]. Because unloaded shortening velocity is set by the rate of cross-bridge detachment, slowed ADP release depresses velocity even where the effect on isometric force is modest, and the two deficits combine into a disproportionate loss of peak power. Treating force and velocity as a single quantity, therefore, understates the contribution of acidosis and overstates how much of the contractile deficit a single isometric twitch can capture.

In exercising humans, the two ions dissociate. In 16 adults performing 60 maximal intermittent isometric knee extensions with simultaneous ^31^P magnetic resonance spectroscopy, the decline in quadriceps twitch force tracked Pi accumulation (r = 0.761, *p* < 0.001) more consistently than hydrogen-ion (H^+^) accumulation, and the magnitude of the fall per unit increase in either metabolite did not differ between the eight women and eight men [14]. That the two ions are not interchangeable in vivo has since been shown directly. When intramuscular H^+^ and Pi were dissociated during intermittent isometric knee extension in 10 men and tracked by ^31^P magnetic resonance spectroscopy, Pi tracked the peripheral component of fatigue while acidosis tracked the central component, consistent with H^+^ acting in the interstitial space on group III/IV afferents rather than on the myofilaments [3]. Rodent work in which contraction frequency was varied under ^31^P nuclear magnetic resonance adds a further feature: peak twitch force did not begin to decline until Pi exceeded a threshold concentration, so the relation between Pi and force loss is not proportional across the whole range but has a breakpoint [1]. This is the strongest available case for a nonlinear Pi–force relationship. A breakpoint is compatible with a defended Pi limit, but demonstrating regulation would require evidence of feedback acting on Pi itself, which has not been reported; the present account therefore neither disputes a Pi limit nor treats one as established.

What the mechanism does not explain is why exercise stops, and this is where the peripheral and the regulatory questions separate. Force loss from H^+^ and Pi is established, is understood at the level of the cross-bridge cycle, and reverses within minutes. Yet athletes tolerate far larger accumulations of both ions when the afferents that sense them are blocked [15], an observation consistent with the interpretation that the accumulation itself is not the terminating event, but rather the tolerability of the signal it generates. Because afferent traffic and contractile impairment have not been recorded simultaneously in exercising humans, this interpretation is supported by, but not demonstrated by, the available evidence. A defended Pi limit and a non-constant twitch decrement are compatible precisely because the twitch decrement sums Pi-driven force loss with processes that are not Pi-driven, as Section 2.2, Section 2.3 and Section 2.4 and Section 3.1 develop.

### 2.2. Sarcolemmal Excitability and K^+^

Acidosis alone does not account for task failure. When pre-exercise muscle acidity was elevated by prior intense arm exercise in seven men, muscle pH at exhaustion was lower (6.65 versus 6.82), and time to exhaustion decreased from 4.67 ± 0.55 to 3.46 ± 0.28 min (*p* < 0.05). Glycogen breakdown was identical, and K^+^ release from the working leg was greater [16]. How K^+^ depresses force is not a simple monotonic relation, and the distinction matters for how the twitch decrement should be read. K^+^ leaves the fiber with each action potential and accumulates in the interstitium and, more consequentially, in the sealed lumen of the transverse tubules, where the surface-to-volume ratio is unfavorable and Na^+^,K^+^-ATPase reclamation is the only route of removal. The resulting depolarization inactivates voltage-gated Na^+^ channels and reduces action-potential amplitude, so fewer action potentials arrive intact at the triadic junction, and Ca^2+^ release falls. Two membrane conductances set the sign of the effect, as an invited review of the ionic regulation of excitability sets out: chloride conductance through skeletal-muscle chloride channel 1 (ClC-1) channels and potassium conductance through ATP-sensitive K^+^ (KATP) channels each carry outward current that shifts the action-potential threshold toward less negative membrane potentials and opposes the inward Na^+^ current during depolarization [17]. For this reason, the change in the K^+^ gradient that exercise produces is by itself too small to be a major cause of force loss; it becomes one only in combination with changes in the Na^+^ gradient and in these two conductances [17].

The consequence is that K^+^ can either potentiate or depress force, depending on metabolic state. At low and moderate intensities, ClC-1 activity falls as contraction begins, which prevents K^+^-induced depression and allows moderate interstitial K^+^ accumulation to potentiate submaximal tetanic force. When ATP demand exceeds supply, both KATP and ClC-1 are activated: KATP lowers the rate at which action potentials are generated, and the rise in chloride conductance lowers the critical extracellular K^+^ concentration at which K^+^ switches from potentiating force to depressing it [17]. The same interstitial K^+^ concentration therefore carries opposite functional meanings on either side of that switch. This is the clearest case in the present review of a mechanism whose weight within the twitch decrement is not merely variable but sign-reversing, and it is one reason a single end-exercise value of the index cannot be interpreted without knowing the metabolic state that produced it.

That K^+^ handling contributes to fatigue in trained humans is suggested pharmacologically: N-acetylcysteine infusion attenuated the decline in maximal Na^+^,K^+^-ATPase activity relative to work performed (*p* < 0.005) and the rise in plasma K^+^, and prolonged time to fatigue at 92% of peak oxygen uptake by 23.8 ± 8.3% in eight well-trained participants (*p* < 0.05) [18,19]. N-acetylcysteine is not specific to K^+^ handling: it also raises muscle cysteine and glutathione availability [19] and may act on redox-sensitive Ca^2+^ release (Section 2.4), so the concurrent changes in Na^+^,K^+^-ATPase activity, and tolerance are an association, not proof that the gain in tolerance ran through K^+^. This mechanism also reverses within minutes, but K^+^ is not among the metabolites that activate these afferents at physiological concentrations [2]; it lowers force without a known afferent stimulus proportional to the effect. One further implication deserves stating, because it bears directly on what is being regulated. The proposed function of the ClC-1 and KATP response is to preserve the remaining ATP, thereby preventing damaging depletion [17]. If that is correct, the quantity protected at the cellular level would be cytosolic ATP rather than force, and the loss of excitability would be the protective mechanism rather than the failure. This is a third candidate for a regulated variable, alongside intramyocellular Pi and the integrated afferent signal, and it reinforces the point made in Section 1: the accounts are not in competition. What follows is that the potentiated-twitch decrement should not be expected to hold a constant value, because it is a downstream consequence of protective responses rather than the protected variable itself.

### 2.3. Sarcoplasmic Reticulum Ca^2+^ Release and Intramyofibrillar Glycogen

In elite cross-country skiers, an exhaustive 1-h race reduced arm glycogen to 31 ± 4% and sarcoplasmic reticulum (SR) Ca^2+^ release rate to 85 ± 2% of pre-race values; withholding carbohydrate for 4 h left these at 29 ± 2% and 77 ± 8%, and the association was with intramyofibrillar glycogen, 10% to 15% of the store, not with total glycogen [20]. In 11 skiers performing four self-paced sprint trials of approximately 4 min, SR Ca^2+^ release rate fell by 17% to 18% in the arms and legs after the first trial (*p* < 0.0001) and further after the fourth trial [21]. The functional consequence is substantial: in 11 men cycling at 75% of peak oxygen uptake, time to exhaustion was 150 (30), 112 (22), and 69 (18) min on high-, mixed-, and low-carbohydrate diets (*p* < 0.001), with intramyofibrillar glycogen in type 1 fibers its strongest correlate [22]. Causality rests on rodent single fibers: fatigued to 30% of initial force, tetanic cytosolic Ca^2+^ fell to 70 ± 4% and 54 ± 4% of initial values, correlating with intramyofibrillar glycogen loss (*p* = 0.0008) rather than with the intermyofibrillar pool [23]. Why a pool amounting to 10% to 15% of the store should matter more than the bulk is a question about location rather than quantity. Glycogen is held in three subcellular compartments, subsarcolemmal, intermyofibrillar, and intramyofibrillar, and depletion is compartment- and fiber-type specific, so individual fibers can be locally emptied while whole-muscle glycogen is only moderately reduced [24]. The traditional explanation that low glycogen limits ATP resynthesis at the whole-muscle level does not fit that pattern. The alternative, developed in a narrative review of glycogen and high-intensity performance, is a direct coupling between glycogen and the steps of excitation–contraction coupling: the intramyofibrillar pool lies closest to the triadic junction and the SR membrane, where glycolytic ATP supply is thought to serve the Ca^2+^ pump and the release channel locally, so its depletion degrades Ca^2+^ handling and membrane excitability before any global energetic shortfall appears [24]. A review of cellular Ca^2+^ handling places the same phenomenon in sequence: myofibrillar impairment dominates early fatigue, reduced SR Ca^2+^ release dominates later stages, and the SR component of prolonged, lower-intensity exercise is the one that tracks glycogen [25]. This is why the mechanism belongs in the composite yet cannot be read off a single twitch. It is a local, compartment-specific failure whose timing differs from that of the metabolite mechanisms in Section 2.1 and Section 2.2.

The human evidence is correlational and not unanimous [26], and this mechanism reverses over hours to days, not minutes.

### 2.4. Reactive Oxygen Species and Ryanodine Receptor Fragmentation

Reactive oxygen species act on the same target, and the effect depends on training status. In recreationally active men, a single session of three to six 30-s all-out cycling bouts, totaling less than 3 min of exercise, fragmented the SR Ca^2+^ release channel, ryanodine receptor 1 (RyR1), and depressed force at 24 h; elite endurance athletes performing the identical session showed no fragmentation. In mouse muscle stimulated to mimic the protocol, fragmentation depended on reactive oxygen and nitrogen species and proceeded through calpain activation, SR Ca^2+^ leak at rest, and impaired Ca^2+^ release [27]. That this mechanism reverses over days rather than minutes follows from the effects of oxidant species on the release channel. A review of cellular Ca^2+^ handling in fatigue sets out that elevated reactive oxygen and nitrogen species produce long-lasting impairment of SR Ca^2+^ release, and therefore a force depression that outlasts the exercise by hours to days, unlike metabolite mechanisms that resolve as Pi and H^+^ are cleared [25]. The same review states the general principle that organizes Section 3: the cellular responses reducing force during intense exercise, reduced Ca^2+^ release and impaired ion pumping among them, act to keep ATP from falling to levels at which rigor cross-bridges form, so declining contractile function is a consequence of protection rather than a failure of it [25]. Whether a rise in these species helps or harms depends on which species is involved and on the magnitude, duration, and location of the rise, together with the antioxidant defenses available [25], which is the most economical account of why the elite athletes above were protected and the recreationally active men were not.

Consistent with a redox contribution confined to conditions where Ca^2+^ and K^+^ handling constrain performance, N-acetylcysteine delayed fatigue at 80% of maximal power but not at 90%, 100%, or 110% (*n* = 7) [28], and did not change mean power in 4-min tests in 11 cyclists (401 ± 44 versus 400 ± 44 W, *p* = 0.39) [29]. The evidence on chronic antioxidant supplementation is limited to a single trial and does not support it as a countermeasure: 1000 mg vitamin C and 235 mg vitamin E daily for 11 weeks in 54 adults left gains in peak oxygen uptake and shuttle-test performance unchanged while abolishing the training-induced rises in cytochrome c oxidase subunit IV and peroxisome proliferator-activated receptor-γ coactivator-1α (PGC-1α) (*p* ≤ 0.03) [30].

### 2.5. Group III/IV Afferent Feedback

Lumbar intrathecal fentanyl blocks the central projection of these afferents without altering motor-nerve conduction or maximal voluntary force. In eight competitive cyclists, blockade increased quadriceps electromyographic activity by 12 ± 3% during the first half of a self-paced 5-km time trial and increased early power output [31]; in seven men cycling at 80% of peak power, it shortened time to exhaustion from 8.7 ± 0.3 to 6.8 ± 0.3 min and accelerated the fall in potentiated twitch force by approximately 67% (*p* < 0.01) [32]. Sampling the muscle during blockade shows the following sequence: the rises in intramuscular Pi, H^+^, and lactate and the fall in phosphocreatine were 47% to 129% larger than under control; the twitch fell 52 ± 2% rather than 31 ± 1% (*p* < 0.001); and the additional decrement was associated with the additional Pi (r^2^ = 0.79) and H^+^ (r^2^ = 0.92) [15]. What these afferents transduce is specific: cultured dorsal root ganglion neurons innervating skeletal muscle respond to combined protons, ATP, and lactate through acid-sensing ion channels (ASICs), P2X and TRPV1 receptors, and far more weakly to any applied alone, in two populations activated at low and high metabolite concentrations [2]. The gain of the loop is not constant. With end-exercise quadriceps fatigue matched at approximately 40%, corticomotoneuronal changes did not differ between trials with intact and blocked afferents (*p* > 0.964, *n* = 9), with voluntary descending drive accounting for most of the change in corticospinal excitability [33]; during 20-min cycling in 13 participants, blockade reduced cardiac output at approximately 50% of peak oxygen uptake (*p* = 0.006) but not at approximately 75% (*p* = 0.741) [34].

The strongest form of the position this review questions deserves to be stated on its own terms, because it is more careful than the version usually summarized. The critical-threshold-of-peripheral-fatigue hypothesis holds that a negative feedback loop protects the exercising limb from severe threats to homeostasis. Its proponents observe that, while the threshold has not been disproven within a given task, it is not generalizable across exercise modalities, and they advance the broader sensory tolerance limit precisely to accommodate that failure: a negative feedback loop summing signals from locomotor muscles, respiratory muscles, organs, and muscles not directly engaged, together with feedforward corollary discharge, to keep voluntary activity tolerable [35]. Three propositions must therefore be kept apart: (i) a universal threshold common to all tasks, which its proponents do not defend and which the between-task differences in Section 3.4 contradict; (ii) a task-specific threshold, a stable end-exercise decrement within one task and condition, which between-task differences do not address and which is examined in Section 3.2; and (iii) the sensory tolerance limit, which is a claim about a summed signal rather than about the twitch. The evidence in Section 3 is assessed against whichever of the three each study actually tested. The present account agrees with the sensory tolerance limit as a construct and departs from it on one point. If the regulated quantity is the summed signal, then the potentiated-twitch decrement, which indexes contractile impairment in a single muscle group and only the metabolite-driven fraction of it, is the wrong measurement to test constancy. The disagreement concerns the measurement, not whether regulation occurs.

### 2.6. Arterial Oxygen Delivery

Clamping arterial oxygen content at four levels in eight trained cyclists increased integrated electromyographic activity by approximately 43% and mean power from 275.0 ± 9.7 to 356.5 ± 12.5 W, while the end-exercise twitch decrement ranged from 33.4% to 35.4% (*p* = 0.19). Under a constant load in the same athletes, time to exhaustion varied fourfold, with a decrement ranging from 31.7% to 34.2% (*p* = 0.44) [36]. Below an arterial oxygen saturation of approximately 70–75%, the relative contribution of central mechanisms increased [37]. Severe hypoxia reduced time to exhaustion by about 70% and produced a greater twitch decrement than time-matched normoxic exercise (−34 ± 4% versus −24 ± 3%, *p* < 0.01) [38]. The mechanistic route is indirect. Arterial oxygen content sets the rate of oxidative ATP resynthesis and therefore the rate at which Pi and H^+^ accumulate for a given power output, which is the quantity the afferents transduce (Section 2.1 and Section 2.5). In these experiments, lowering oxygen delivery accelerated that accumulation while the end-exercise decrement changed little, as shown by a fourfold variation in time to exhaustion for a 2.5-percentage-point difference in decrement [36]. This dissociation is also the clearest instance of the non-generalizability across modalities that proponents of the threshold hypothesis themselves concede [35]. Within this task, therefore, oxygen delivery altered how quickly contractile impairment developed more than the value at which exercise ended, the pattern a task-specific threshold predicts; it does not bear on whether that value is common across tasks.

### 2.7. Respiratory-Muscle Work

Inhibitory feedback is not confined to the working limb. Reducing inspiratory-muscle force output by 56% with a proportional-assist ventilator, at identical external power and duration, attenuated the quadriceps twitch decrement from −28 ± 5% to −20 ± 5%; increasing inspiratory-muscle force output by 80% with resistive loads exacerbated the decrement from −12 ± 8% to −20 ± 7% (both *p* < 0.05, *n* = 8) [39]. Respiratory fatigue itself accrues progressively rather than at a threshold: cycling in the severe-intensity domain reduced gastric and diaphragm twitch pressures by 11.9 ± 8.2% and 9.5 ± 9.2% at half the tolerable duration and by 30.3 ± 15.6% and 22.4 ± 12.5% at task failure (all *p* < 0.05, *n* = 10) [40]. The pathway is both vascular and neural. Metabolite accumulation in fatiguing inspiratory and expiratory muscles activates their own group III/IV afferents, thereby increasing sympathetic vasoconstrictor outflow and redistributing cardiac output; because flow to the respiratory pump comes at the expense of the working limb, the locomotor muscle receives less oxygen and accumulates metabolites more quickly. A synthesis of this literature describes a two-way street of sympathetic vasoconstrictor activity arising from both limb and respiratory metaboreceptors, constraining blood flow and oxygen transport, thereby promoting fatigue in both sets of muscles [41]. Two consequences follow for the index. The respiratory contribution enters the quadriceps twitch decrement without originating in the quadriceps, and the feedback proposed to terminate exercise would sum across muscle groups the measurement never samples. A limit defined solely by the locomotor muscle is therefore unlikely to be general.

### 2.8. Substrate Availability

Beyond its effect on SR Ca^2+^ release (Section 2.3), substrate availability shifts the intensity boundary itself. In 16 endurance-trained cyclists, 3 h at 95% of the gas exchange threshold lowered critical power from 277 ± 27 to 236 ± 30 W on water, and carbohydrate attenuated that loss in a dose-dependent manner, to 257 ± 28 and 266 ± 29 W at 60 and 120 g·h^−1^ (*p* < 0.05), without affecting W′, the finite work capacity available above critical power [42]. The mechanism linking substrate to the intensity boundary is the compartmental one described in Section 2.3 rather than a global energetic one. Critical power depends on the capacity to resynthesize ATP oxidatively at a sustainable rate, and the fiber-type- and compartment-specific depletion accompanying prolonged exercise reduces that capacity in the fibers recruited at those intensities well before whole-muscle glycogen approaches exhaustion [24]. That W′ was unaffected while critical power fell is consistent with this interpretation, since W′ indexes a finite work capacity above the boundary rather than the position of the boundary itself. Mode determines which contractile variable is affected: after 60-km self-paced cycling in the heat in 10 well-trained cyclists, the response was dominated by slowed twitch relaxation, with no condition effects on maximal voluntary contraction, voluntary activation, or M-wave amplitude (all *p* ≥ 0.335), and carbohydrate did not attenuate it [43].

### 2.9. Central and Perceptual Mechanisms

Voluntary activation declines during high-intensity aerobic exercise, with the magnitude varying by task: across 4-, 20-, and 40-km time trials, it fell 7%, 11%, and 10% with motor-nerve stimulation and 6%, 12%, and 10% with cortical stimulation [4]; under constant load, it fell 2.7 ± 2.2% after exercise at peak aerobic power but 9.0 ± 4.7% at the respiratory compensation point (*p* < 0.01) [44]. Perceptual and cognitive load affect the same output without changing muscle activity: 30 min of a mentally fatiguing task reduced W′ from 29.3 ± 6.3 to 22.8 ± 4.5 kJ without altering critical power in 11 trained cyclists, with lower blood lactate, so exercise ended before W′ was expended [45]; and 90 min of a cognitive task cut time to exhaustion at 80% of peak power from 754 ± 339 to 640 ± 316 s (*p* = 0.003) with unchanged cardiorespiratory responses but higher perceived effort (*p* = 0.007) [46].

That a cognitive task with no muscular component can shorten tolerance requires a mechanism in the brain rather than in the muscle. A review of brain neurotransmission in exercise-induced and mental fatigue attributes the effect to neuromodulator changes in the prefrontal and anterior cingulate cortex, with dopamine and adenosine as the principal candidates, and separately reports that noradrenergic activity hastens central fatigue during prolonged exercise, paralleling a faster rise in the rating of perceived exertion [47]. Perceived effort is therefore not solely a readout of contractile state; it has additional determinants that can shift the termination point independently. This mechanism, in the review, is the furthest removed from the potentiated twitch, and it is the plainest reason the index cannot be treated as sufficient: two of the manipulations in this section shorten exercise tolerance without changing muscle activity at all.

## 3. Discussion

### 3.1. What the Potentiated Twitch Actually Measures

**Figure 1:**
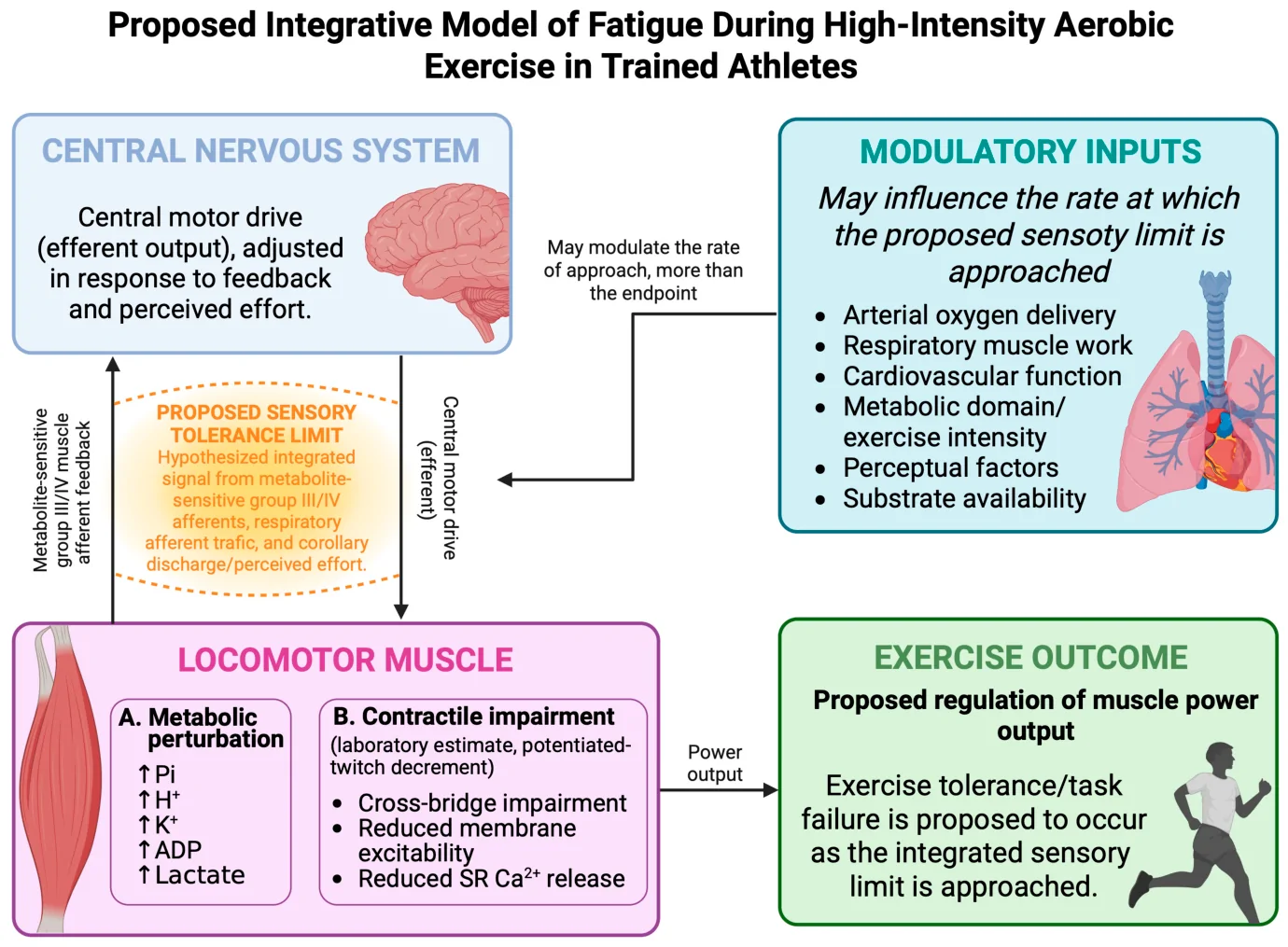
Proposed integrative model (hypothetical) of the relationship between the afferent signal, its laboratory estimate, and the systems that set the rate of accumulation. Group III/IV muscle afferents respond to protons, ATP, and lactate via acid-sensing ion channels, P2X, and TRPV1 receptors, and their central projection reduces motor output. Blocking that projection with lumbar intrathecal fentanyl raises motor output and accelerates the decline in contractile force. We propose that exercise ends when this feedback, summed with afferent traffic from the respiratory muscles and the corollary discharge accompanying the motor command, becomes intolerable; this termination rule is inferred from converging observations and has not been demonstrated directly. The quantity proposed to be regulated is that summed signal; the potentiated-twitch decrement estimates it only in proportion to the metabolite-driven share of contractile impairment, because the afferents sense the metabolites and not the impairment itself. The afferent agonists [2] and the effects of fentanyl blockade [15,31,32] rest on direct experiments; the summation across sources and the termination rule are inferences. The diagram is schematic: no values are plotted, and no fixed end-exercise threshold of contractile impairment is depicted or implied, consistent with the argument developed in Section 3.1, Section 3.2, Section 3.3, Section 3.4, Section 3.5, Section 3.6, Section 3.7 and Section 3.8. In the studies reviewed, arterial oxygen delivery, respiratory muscle work, substrate availability, and perceived effort changed the rate at which the signal accumulates more than its end-exercise value.

The first three mechanisms are not alternatives; they are summed by the index used to quantify peripheral fatigue, and they differ in reversibility by three orders of magnitude (Table 1). Of the three, only cross-bridge impairment is driven by stimuli that are known agonists of the afferents that carry the feedback; these neurons respond to protons, ATP, and lactate [2]. They do not sense twitch force, glycogen content, or SR Ca^2+^ release rate, and no experiment has shown that the magnitude of cross-bridge impairment itself is transduced; rather, the same metabolites produce both the impairment and the afferent discharge. Whether glycogen depletion or reduced Ca^2+^ release alters afferent feedback indirectly has not been tested, so the absence of a known agonist is not evidence of no influence. The twitch decrement can therefore serve as a proxy for the afferent signal only insofar as it is metabolite-driven (Figure 1). We propose that this proportion is not fixed. In short, at high cycling intensity, it is large, so the proxy tracks the signal [10,14]. As duration increases, SR Ca^2+^-release impairment contributes more [20,21,22]. An added, unsensed component would by itself enlarge the decrement, not reduce it; the proposed explanation for the smaller decrement in longer cycling [4,44] is that a larger share of the terminating signal then arises from perceived effort and from non-locomotor sources (Section 2.7 and Section 2.9), so exercise ends before the locomotor metabolite-driven component reaches the value seen in short exercise, while the Ca^2+^-release component adds to the measured decrement without contributing to termination. The composition of the decrement therefore changes with duration, and its total depends on the balance of these terms. This is a hypothesis: no study has quantitatively separated the contributions of the individual mechanisms to the twitch decrement, and the proposed sequence has not been tested directly. Training status changes the composition itself [27], and active muscle mass changes the systemic load per unit of local impairment [6]. A composite index measured at one moment after exercise would behave as a regulated variable only if the weights of its components were constant or their sum were itself sensed; the evidence reviewed here indicates neither, although the partitioning that would settle the point has not been done.

### 3.2. Evidence for a Common End-Exercise Threshold

Three findings are consistent with a task-specific threshold within their respective conditions. Pre-fatiguing the quadriceps by 36% before a 5-km time trial reduced central motor output by 23% and mean power by 14%, yet the end-exercise decrement was 35% to 37% across all conditions (*p* = 0.35, *n* = 8) [48]. Clamping arterial oxygen content markedly altered power and time to exhaustion, with the decrement ranging from 31.7% to 35.4% (Section 2.6) [36]. During intermittent isometric contractions above critical torque in nine men, task failure occurred when maximal voluntary torque fell to the target torque (88.7 ± 4.3 versus 89.3 ± 8.8 N·m, *p* = 0.94) [49]. All three findings rest on failure to reject a null hypothesis in samples of seven to nine, which is evidence of no detected difference rather than evidence of equivalence, and on a measurement whose properties are examined next. The same standard applies in the other direction: several studies in Section 3.4 also have samples of 8 to 13, and a significant between-task difference there shows that the decrement differed, not how precisely; the magnitudes and, where reported, their dispersion are therefore given alongside *p* values, and none of these studies tested equivalence formally.

### 3.3. The Index Recovers at Task-Dependent Rates

Peripheral fatigue is inferred from a stimulus delivered after exercise stops. Assessing neuromuscular function within 5 s of cessation and again at 1, 2, 4, and 8 min in 10 participants showed that evoked torque fell to 33–68% of baseline, depending on the stimulation method, with substantial recovery in the first 1–2 min [8]. The decisive comparison used self-paced trials of three durations in 12 trained participants; evoked forces, including the ratio of low- to high-frequency doublets, were similar across the 3-, 10-, and 40-min trials at cessation but had recovered more after the 3-min trial when measured 1 or 2 min later [9]. A delay of 1–2 min therefore creates between-task differences that did not exist at cessation and can conceal differences that did; studies on both sides report twitches obtained after such delays.

### 3.4. Direct Tests Across Duration, Prior Exercise, Muscle Mass and Mode

Where a threshold common across tasks has been tested rather than inferred, the end-exercise decrement has differed between conditions (Table S1). These tests address a universal threshold; they do not test whether a stable value exists within one task and condition, and several rest on samples of 8 to 13, so the differences below are reported with their magnitudes and are not a verdict on the task-specific hypothesis.

Duration: Across self-paced isokinetic knee-extension trials of 3, 10, and 40 min in 12 trained participants, evoked peak force declined more in the 40-min trial than in the 3-min trial (−42 ± 15% versus −37 ± 13%, *p* < 0.05), with no common threshold across all three [50]. In 13 well-trained cyclists completing 4-, 20-, and 40-km time trials, the potentiated twitch fell by 40%, 31%, and 29% [4]; under constant load in 12 well-trained cyclists exercising for approximately 3, 11, and 42 min, the decrements were −33 ± 9%, −16 ± 9%, and −11 ± 9% (*p* < 0.001 between the shortest and each of the others) [44].

Prior exercise: Prior arm exercise shortened subsequent cycling at a fixed 85% of peak power by 38% (4.33 ± 1.10 versus 7.46 ± 2.79 min, *p* < 0.001) and reduced, rather than preserved, the end-exercise twitch decrement (−26 ± 10% versus −38 ± 13%, *p* = 0.004). The shortfall was predicted by the rate of increase in limb discomfort (r = −0.72 with cycling time, *p* = 0.045), not by contractile failure [7].

Active muscle mass: Single-leg knee extension to exhaustion produced a potentiated-twitch decrement of −44 ± 6% and a maximal voluntary contraction decrement of −25 ± 3%, compared with −33 ± 7% and −12 ± 3% for double-leg exercise at the same relative intensity (*n* = 8) [6]. In 11 men, muscle groups of different size at 15% above critical torque showed peripheral fatigue at task failure of −63.8 ± 20.9% for the knee extensors versus −29.0 ± 14.5% for the handgrip (*p* < 0.001), and central fatigue differed likewise (−14.9 ± 12.4% versus −7.66 ± 5.23%, *p* < 0.001), despite similar declines in maximal torque and similar tolerance [51]. In incremental cycling, the same asymmetry appeared within one locomotor mode: potentiated twitch force fell 45.7 ± 18.8% after single-leg and 30.0 ± 14.0% after double-leg cycling (*p* = 0.049), with no difference in time to task failure, maximal voluntary contraction, or voluntary activation [52].

Exercise mode: In 32 male trail runners across five field races, knee-extensor twitch torque decreased more after races longer than 100 km than after races shorter than 60 km (−28 ± 14% versus −14 ± 10%, *p* = 0.005), and race distance correlated with the magnitude of the decrement (ρ = −0.521, *p* ≤ 0.003) [5]. The duration effect in running is therefore opposite in sign to that in cycling [4,44].

### 3.5. Central and Peripheral Contributions Move in Opposite Directions

Central deficits do not behave as a residual left after a peripheral ceiling is reached. Across the 4-, 20-, and 40-km time trials, voluntary activation fell progressively as the peripheral decrement decreased [4], and under constant load, the central deficit was largest where the peripheral decrement was smallest (Section 2.9) [44]. A peripheral limit common across these tasks predicts the opposite pattern.

### 3.6. What the Evidence Supports

Motor output is regulated; what the evidence assembled here shows directly is that the end-exercise twitch decrement is not constant across tasks. We propose, as an interpretation compatible with that evidence rather than a demonstrated regulatory variable, that the regulated quantity is better described as the sum of the afferent and effort signals than as the contractile index used to estimate that sum. The two coincide when metabolite accumulation predominates, the condition in which a ceiling was first reported [36,48,49], and they diverge as the Ca^2+^-dependent share grows [4,44], when eccentric loading adds another contributor [5], or when the systemic load per unit of local impairment changes [6,51,52]. Oxygen delivery, respiratory muscle work, substrate availability, and perceived effort in the studies reviewed altered the rate at which the signal accumulates, rather than a terminal value [36,39,40,42,45,46].

### 3.7. Relation to a Concurrently Proposed Framework

A framework published while this review was in preparation reaches a similar starting point from the nutritional side: task failure reflects the convergence of intramuscular, neural, perceptual, and behavioral boundaries rather than a single mechanism, with Pi impairing cross-bridge function, Ca^2+^ sensitivity, and excitation–contraction coupling, and group III/IV feedback constraining motor output and voluntary activation [53]. It is cited for positioning, not as evidence. The accounts diverge on one testable point: making Pi the common cause of all three effects implies that the entire contractile decrement is metabosensed, thereby keeping the twitch a valid proxy. The measurements above identify a component whose proximate cause is not a known afferent agonist. After less than 3 min of all-out cycling, RyR1 fragmentation and depressed force were present at 24 h, hours after phosphate had resolved, and were mediated by a redox pathway; elite athletes performing the identical session showed no fragmentation [27] (Table 1), and no phosphate difference is available to explain the training-status effect.

### 3.8. Measurements That Would Falsify This Account

**Figure 2:**
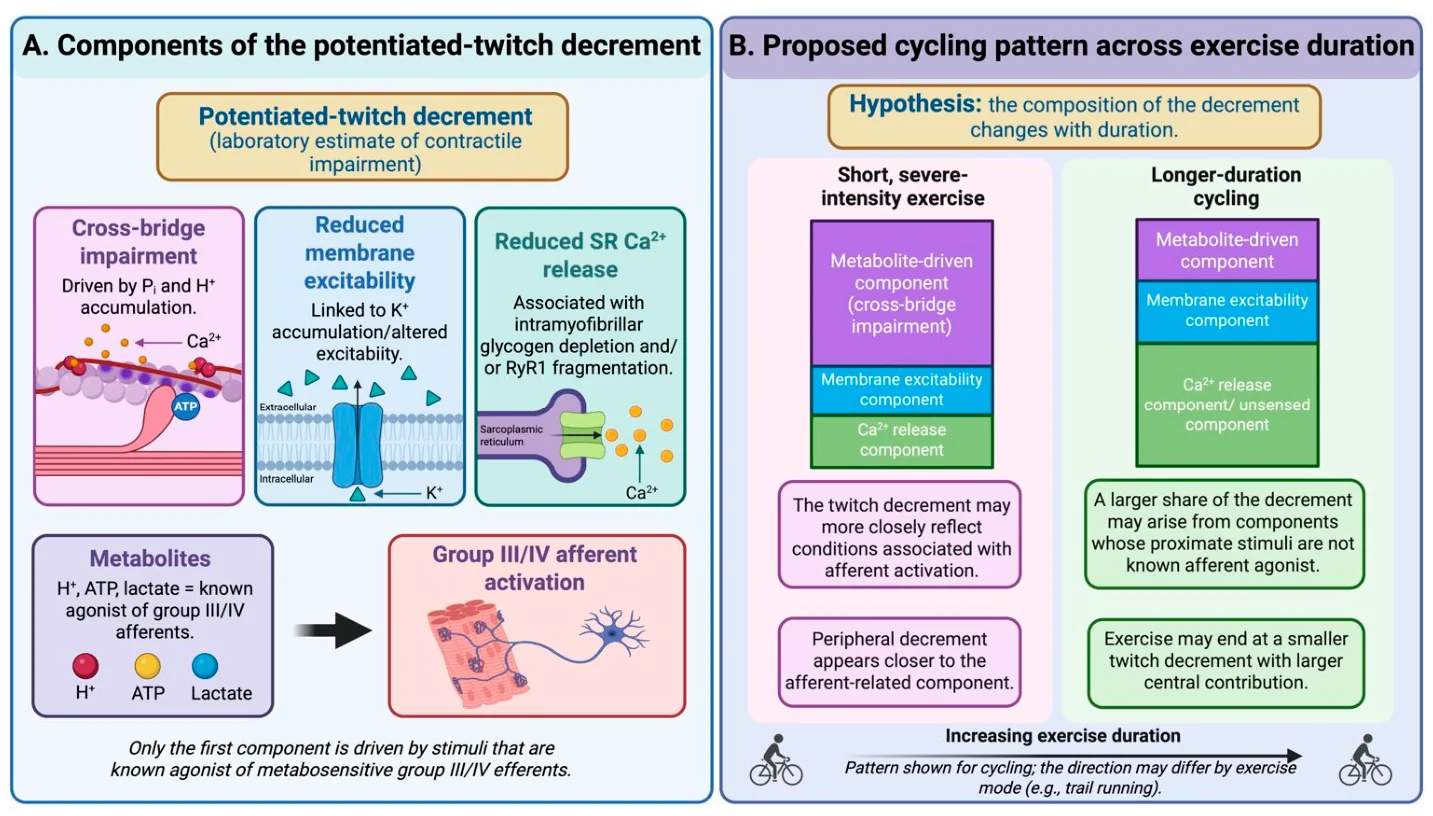
Proposed explanation (hypothesis) of why the end-exercise twitch decrement is not constant. Panel (**A**): Contractile impairment, measured as potentiated twitch force, reflects cross-bridge impairment by inorganic phosphate and hydrogen ions, reduced sarcolemmal excitability from K^+^ accumulation, and reduced sarcoplasmic reticulum Ca^2+^ release from intramyofibrillar glycogen depletion and ryanodine receptor fragmentation. Only the first is driven by metabolites that are known agonists of muscle afferents; the afferents do not sense contractile impairment itself. Panel (**B**): In short, severe-intensity exercise, the metabolite component predominates, so the twitch decrement tracks the afferent signal and appears to converge on a common value; as duration increases, the Ca^2+^-release component is proposed to contribute a larger share whose proximate stimulus is not sensed by these afferents, so exercise would end at a smaller twitch decrement with a larger central deficit. Panel B depicts the pattern reported in cycling [4,44]; in trail running, the peripheral decrement increased with race distance [5], so the direction of the duration effect is mode-dependent and the panel is not general. Panel A is schematic, and its proportions were not estimated from data; the curves in Panel B are qualitative and illustrative, and no value shown is a quantitative estimate.

Four predictions follow, each of which is testable with existing methods (Figure 2). First, assessing neuromuscular function within 5 s of cessation should reduce between-task differences in the end-exercise decrement relative to assessment at 1 to 2 min, and residual differences should reflect mechanistic composition rather than duration [8,9]. Second, lowering muscle glycogen, which reduces SR Ca^2+^ release with comparatively small changes in Pi and H^+^ [20,26], should dissociate the twitch decrement from perceived effort and from afferent-dependent restraint of motor output; an account based on metabolites alone predicts they move together. Third, N-acetylcysteine should extend tolerance where K^+^ and Ca^2+^ handling limit performance, rather than where cross-bridge impairment does [19,28,29]. Fourth, because SR Ca^2+^ release is resistant to disruption in trained athletes [27] and four weeks of training increased its rate by 9% in 19 trained athletes (*p* = 0.009) [21], the end-exercise decrement should be more consistent across durations in trained than in untrained participants. The one relevant trial is not yet supportive: five weeks of sprint interval training in 21 recreationally active men increased power at the respiratory compensation point by 11.0% compared with −0.7% in controls (*p* < 0.01), but left performance fatigability unchanged [54]. It measured a single duration, so it does not test the prediction; however, a performance gain does not necessarily shift the fatigue endpoint.

The account would be refuted by any of the following. First, if decrements measured within 5 s of cessation converged on a common value across durations, modes, and active muscle masses, a defended peripheral threshold would be supported, and the composite account contradicted. Second, if glycogen depletion lowered the twitch decrement and perceived effort and motor output fell in proportion, the Ca^2+^-release component would be behaving as a sensed component, contrary to the account. Third, if the twitch decrement at task failure remained the same when the metabolite-driven and Ca^2+^-driven fractions were shifted experimentally at matched intensity, the twitch itself would be behaving as the regulated variable. Conversely, no observation about the twitch alone distinguishes this account from the sensory tolerance limit, with which it shares the summed signal; the two differ only in whether the twitch is treated as a valid proxy for that signal, and that is what the second and third tests examine.

## 4. Materials and Methods

To ensure transparency and, as far as possible, reduce bias and misinformation in this type of review, we present the methodology used to gather the information.

### 4.1. Design and Reporting

This narrative, mechanism-oriented review was prepared and reported in accordance with SANRA (Scale for the Assessment of Narrative Review Articles). It is not a systematic review: it was not registered, no protocol was published, and no formal risk-of-bias assessment or quantitative synthesis was conducted. The principal Tier 1 studies were instead critically appraised for sample size, design, training status, method and timing of neuromuscular assessment, and main methodological limitations (Table S5), so that readers can judge the strength of each line of evidence. Evidence was purposively selected to address a single question: whether the potentiated-twitch decrement behaves as a regulated variable. The conclusions are framed accordingly, as an interpretation of converging measurements rather than as an estimate of an effect.

### 4.2. Information Sources and Search Strategy

PubMed/MEDLINE, Scopus, and Web of Science were searched from inception to 12 August 2026, with backward and forward citation tracking of every retained report. No language, date, or design limits were applied at the search stage. The PubMed/MEDLINE strategy was: (“muscle fatigue”[MeSH] OR fatigue[tiab] OR fatigability[tiab] OR “task failure”[tiab] OR “time to exhaustion”[tiab]) AND (“physical endurance”[MeSH] OR exercise[MeSH] OR endurance[tiab] OR “high-intensity”[tiab] OR “time trial”[tiab] OR cycling[tiab] OR running[tiab]) AND (“afferent pathways”[MeSH] OR “group III”[tiab] OR “group IV”[tiab] OR “voluntary activation”[tiab] OR “central fatigue”[tiab] OR twitch[tiab] OR “sarcoplasmic reticulum”[MeSH] OR “calcium release”[tiab] OR “ryanodine receptor”[tiab] OR glycogen[MeSH] OR “reactive oxygen species”[MeSH] OR potassium[MeSH] OR phosphates[MeSH] OR “hydrogen-ion concentration”[MeSH] OR “oxygen consumption”[MeSH] OR “respiratory muscles”[MeSH] OR “perceived exertion”[tiab]). The strings adapted to Scopus (TITLE-ABS-KEY) and Web of Science (TS=) syntax, with the records returned by each, are given in Table S2. Because the strategy names the candidate mechanisms, it was designed to retrieve evidence on a defined set of candidates relevant to the question posed, not to identify comprehensively every mechanism of fatigue in high-intensity exercise; the title, objective, and conclusions are framed accordingly.

### 4.3. Eligibility Criteria

Because the question spans levels of biological organization, eligibility was defined in three tiers, and every citation in the text belongs to one of them; the tier, species or preparation, and training status of every cited report are listed in Table S4.

Tier 1, primary evidence: Original controlled studies in healthy adult humans that quantify a neuromuscular, cardiorespiratory, metabolic, or perceptual index of fatigue during or immediately after whole-body or single-joint exercise. Trained, well-trained, elite, and recreationally active samples were all eligible because the training-status contrast is itself one of the phenomena under review (Section 2.4 and Section 3.8). Training status is reported as characterized in each source and is not comparable across studies.

Tier 2, mechanistic corroboration: Experiments on permeabilized or intact human fibers, on rodent muscle or single fibers, and on isolated actomyosin preparations, including single-molecule and mini-ensemble laser-trap assays, are eligible only when measurements cannot be obtained in exercising humans, for example, cytosolic Ca^2+^ during fatigue or the causal role of a redox pathway. Tier 2 sources are identified as such at each mention and never make a claim about exercising athletes on their own.

Tier 3, mechanism and positioning: Reviews, editorials, and conceptual frameworks. These are used to locate primary sources, to position the present account (Section 3.7), and, where a mechanism has been established across a body of work too large to cite primarily, and no single primary report states it, to source the mechanistic account itself. A Tier 3 source is identified as a review at each mention and is never used to support a quantitative result, a magnitude, or a claim about a particular sample; those rest only on Tier 1 or Tier 2 evidence.

Samples outside the trained-athlete range, including recreationally active and older participants, were eligible where they provided the only retrieved test of a mechanism; each such citation is identified in the text and treated as a boundary condition rather than as evidence about trained athletes (Section 5.1). Case reports, conference abstracts, theses, non-peer-reviewed preprints, and studies reporting performance without any measured index of fatigue were excluded.

### 4.4. Study Selection

Two authors (A.R.-J. and M.R.-V.) screened titles and abstracts independently against the tiered criteria, then assessed the full texts of retained records. Disagreements were resolved by consensus, and when consensus was not reached, by a third author (M.A.H.-L.). Records identified per database, duplicates removed, records screened, full texts assessed, reports excluded with reasons, and reports included are reported in Table S3, where the counts reconcile arithmetically. Because the review is narrative and its selection was purposive, these counts describe the records considered for a defined question rather than an exhaustive census of the literature on muscle fatigue.

### 4.5. Selection of Candidate Mechanisms and of Their Supporting Studies

Candidate mechanisms were not selected by citation frequency. A mechanism was retained for Section 2 only if it met three selection conditions. Because no protocol was registered or published, these conditions cannot be shown to have been established prospectively and are described as the criteria used rather than as pre-specified: (i) at least one Tier 1 experiment reported a change in the mechanism during or after high-intensity aerobic exercise in humans; (ii) at least one experiment manipulated the mechanism, pharmacologically, nutritionally, or by altering oxygen delivery, respiratory-muscle work, or active muscle mass, and reported the consequence for exercise tolerance or for a measured fatigue index; and (iii) its timescale of reversal and whether it is transduced by metabosensitive afferents could both be established from the retrieved evidence, since the argument in Section 3.1 requires both. Mechanisms for which a change was reported but no manipulation was retrieved did not meet condition (ii) and are not discussed. Within each retained mechanism, the studies cited are the Tier 1 experiments that manipulated it, together with the Tier 2 experiment that establishes causality where the Tier 1 evidence is correlational; where retrieved reports conflicted in direction, both are reported (Section 2.6 and Section 3.4). Every quantitative result cited in this review is traceable to an original research report, so each magnitude can be checked against a measurement rather than an interpretation. Reviews are cited in two circumstances only: to position the present account, which applies to a concurrently published framework discussed in Section 3.7, and to state a mechanism that is established across a literature too large to cite primarily and that no single primary report sets out in full, which applies to the ionic regulation of sarcolemmal excitability in Section 2.2. In both circumstances, the source is identified as a review at the point of citation and carries no numerical claim.

## 5. Conclusions

Fatigue during high-intensity aerobic exercise in trained and recreationally active adults is unlikely to arise from a single primary mechanism. Cross-bridge impairment, loss of sarcolemmal excitability, impaired SR Ca^2+^ release, redox damage to the release channel, afferent feedback, oxygen delivery, respiratory-muscle work, substrate availability, and perceptual load each have direct experimental support and differ in how quickly they reverse and whether they are sensed. The measurements reported in this review do not support a fixed end-exercise value of peripheral fatigue common across tasks, although a stable value within a single task and condition remains compatible with the data: direct tests find different decrements across duration, prior exercise, active muscle mass, and mode [4,5,6,7,44,50,51,52], and the index itself recovers at task-dependent rates [8,9]. Treating it as a composite whose weights change with intensity, duration, mode, and training status accounts for these results without discarding the afferent loop and specifies what would refute it. Given the purposive selection of evidence, the absence of formal risk-of-bias appraisal, and the reliance on inference from converging rather than simultaneous measurements, this composite account should be read as a hypothesis to be tested by the experiments proposed in Section 3.8, not as an established replacement for the fatigue-ceiling model.

### 5.1. Limitations

Evidence selection was purposive and lacked the bias-assessed coverage of a systematic review. The search is reported reproducibly (Section 4.2) but was not designed to be exhaustive, so eligible reports may have been missed, and no included study was formally appraised for risk of bias. Part of the evidence base also lies outside the trained-athlete population, in recreationally active and older participants, and part is Tier 2, from permeabilized human fibers and rodent muscle (Section 4.3); conclusions about trained athletes therefore rest in part on extrapolation across training status and species. Six studies supporting the afferent loop and the oxygenation experiments come from one group [15,31,32,36,38,48], and four negative tests come from another [4,8,9,50], so part of the disagreement may reflect methodology rather than physiology. Participants were predominantly male, although the fall in twitch force per unit of metabolite accumulation did not differ between eight women and eight men [14], and neither performance nor its determinants differed between male and female cyclists during 4-km time trials [55]. Two muscle-mass replications were obtained outside the trained-athlete population [51,52]. The link between intramyofibrillar glycogen and Ca^2+^ release rests on rodent single-fiber measurements [23]. The search strategy named the candidate mechanisms, so mechanisms outside that set could not be retrieved, and the nine mechanisms reviewed are candidates relevant to the question posed, not an exhaustive account of fatigue in high-intensity exercise. The principal Tier 1 studies were critically appraised (Table S5) but not formally rated for risk of bias, and most have samples of 7 to 16, so both the null results in Section 3.2 and the between-task differences in Section 3.4 are imprecise. The included tasks range from brief all-out sprints and single-joint isometric contractions to ultra-distance running and several hours of moderate-intensity exercise; they are not interchangeable fatigue tasks, and comparisons across them are qualitative. Finally, the central proposition here is an inference from converging measurements, not a direct observation.

### 5.2. Practical Implications

1. Interventions that slow metabolite accumulation, such as pacing, improved oxygen delivery, and reduced respiratory muscle work, extended tolerance in the studies reviewed; however, the evidence retrieved here does not identify a fixed peripheral threshold common across tasks that training could target.
2. A mechanistically informed hypothesis, not an established hierarchy, is that carbohydrate availability acts on the SR Ca^2+^-release component in efforts long enough for that component to limit force and contributes less through this pathway in efforts of a few minutes; carbohydrate also acts through routes outside this pathway, and the studies reviewed did not compare its efficacy across durations.
3. In one 11-week trial in trained and untrained adults, high-dose vitamin C and E abolished the training-induced rise in mitochondrial markers without improving performance [30]; this single finding does not support chronic high-dose antioxidant supplementation as a countermeasure for the fatigue mechanisms reviewed here, and the question lies outside the main scope of this review.
4. Neuromuscular function should be measured within seconds of cessation; a 1- to 2-min delay changes which task appears to have produced more peripheral fatigue.

## Figures and Tables

**Table 1:** The three processes, as summarized by the potentiated-twitch index, and how they differ. Only the first is driven by stimuli that are known agonists of metabosensitive group III/IV afferents, so only the first can plausibly serve as a proxy for the afferent signal; the afferents sense the metabolites, not the contractile impairment itself.

Process	Proximate Cause	Principal Evidence	Reversal	Proximate Stimulus Sensed by Afferents?	Dominant When
Cross-bridge impairment	Accumulation of inorganic phosphate and hydrogen ions; Pi reverses the power stroke and favors low-force actomyosin states, depressing isometric force; H^+^ slows ADP release and the weak-to-strong binding transition, depressing shortening velocity and peak power; Pi also depresses SR Ca^2+^ release	254 permeabilized myofibers from 12 men at pH 6.2 and 30 mM Pi; in vivo ^31^P spectroscopy, twitch loss vs. Pi r = 0.761 (*n* = 16); laser-trap mini-ensembles, acidosis reduces force-generating capacity by 20%; in vivo H^+^/Pi dissociation (*n* = 10) separates the peripheral from the central component	Minutes	Indirectly—its proximate causes, protons and ATP, are direct agonists of ASICs, P2X and TRPV1 on muscle afferents; the impairment itself is not sensed	Short, severe-intensity exercise
Reduced membrane excitability	Interstitial and t-tubular K^+^ accumulation exceeding Na^+^,K^+^-ATPase reclamation; sarcolemmal depolarization and reduced action-potential propagation	Prior-arm-exercise acidosis: greater K^+^ release, glycogenolysis unchanged (*n* = 7); N-acetylcysteine attenuates the fall in Na^+^,K^+^-ATPase activity and delays fatigue by 23.8 ± 8.3% (*n* = 8)	Minutes	Partly—co-released metabolites are sensed, but K^+^ itself is not the principal afferent stimulus	Prolonged submaximal exercise with high ventilatory and K^+^ load
Reduced SR Ca^2+^ release	Depletion of the intramyofibrillar glycogen pool (10–15% of total glycogen); reactive oxygen species-dependent RyR1 fragmentation, calpain activation and SR Ca^2+^ leak	Elite skiers: glycogen 31 ± 4%, release rate 85 ± 2% of initial; release rate fell 17–18% after one 4-min trial (*n* = 11); RyR1 fragmentation after one session in recreational but not elite athletes	Hours to days	No known agonist—glycogen content and Ca^2+^ release rate are not sensed; an indirect influence has not been tested	Prolonged exercise; recreationally active more than elite athletes

## Data Availability

No new data were created or analyzed in this study. Data sharing is not applicable to this article.

## Supplementary Materials

The following supporting information can be downloaded at https://www.mdpi.com/article/10.3390/muscles5040074/s1, Table S1: Direct tests of a common end-exercise peripheral fatigue threshold across duration, prior exercise, active muscle mass, and exercise mode; Table S2: Search strings adapted to PubMed/MEDLINE, Scopus, and Web of Science and records returned by each database; Table S3: Records identified, screened, assessed, excluded with reasons, and included; Table S4: Evidence tier, species or experimental preparations, and training status of reports included in the analysis; Table S5: Critical appraisal of the principal Tier 1 studies (sample size, design, training status, method and timing of neuromuscular assessment, main limitations).
