# Supplementary material for: Is the End-Exercise Potentiated-Twitch Decrement a Regulated Variable? A Mechanism-Oriented Review of Muscle Fatigue During High-Intensity Aerobic Exercise
Source: Muscles. 2026 Oct 9;5(4):74. doi: 10.3390/muscles5040074 (PMC13692533; doi:10.3390/muscles5040074)
Supplement: Supplementary file 1 [file muscles-05-00074-s001.zip › Table S1.pdf]

Table S1. Direct tests of a common end-exercise peripheral fatigue threshold across duration, prior exer-cise, active muscle mass, and exercise mode.

| Study | Factor tested | Participants | Comparison | End-exercise peripheral fatigue measure and outcome | Main observation |
|---|---|---|---|---|---|
| Thomas et al., 2015 [4] | Exercise duration/intensity | 13 well-trained male cyclist; self paced stimulated cycling time trials | 4, 20, and 40 km | Potentiated twitch decrement: -40±20%, -31±17%, and -29±18%, respectively. | Peripheral fatigue was greater after 4 km simulated time trial. Central fatigue showed the opposite pattern, with larger reduction in voluntary activation after longer trials. |
| Temesi et al., 2021 [5] | Exercise mode/ race distance | 32 experienced male trail runners | LONG >100 km vs SHORT <60 km | Knee-extensor potentiated twitch torque decreased -28±14% in LONG vs -14±10% in SHORT; $p = 0.005$. | Race distance was negatively correlated with the change in knee-extensor twitch torque, indicating a larger loss with increacing race distance. |
| Rossman et al., 2014 [6] | Active muscle mass | 8 recreationally active healthy males | Single-leg vs double-leg knee-extensor exercise | Potentiated quadriceps twitch decrement -44±6% vs -33±7%, respectively. | Single-leg exercise, involving less active muscle mass, produced significantly greater peripheral quadriceots fatigue than double.ked exercise. |
| Johnson et al., 2015 [7] | Prior upper-body exercise | 8 healthy, moderate trained men; fixed-work-rate cycling al 85% Wpeak | CYC vs ARM-CYC (cycling after prior high-intensity arm-cranking) | Potentiated quadriceps twitch force decreased -38±13% after CYC vs -26±10% after ARM-CYC; $p = 0.004$. | The rate of increase in leg-discomfort RPE was faser in ARM-CYC and correlated with the reduction in cycling time. |
| Thomas et al., 2016 [44] | Exercise duration/intensity | 12 well-trained male cyclist; randomized crossover constant-load cycling to the limit of tolerance | S+, S-, and RCP trials | Potentiated twitch decrement: -33±9%, -16±9% and -11±9%, respectively. | Peripheral fatigue was greater after the highest-intensity, shortest-duration trial. The pattern for central fatigue was reversed with greater central fatigue at the lowe intensity, londer-duration condition. |
| Froyd et al., 2016 [50] | Exercise duration/intensity | 12 trained participants (11 men, 1 woman); repetitive self-paced concentric right knee-extension time trial on an isokinetic dynamometer | 3-, 10-, and 40-min | For PS100 evoked peak force post-values were 63±13%, 62±12%, and 58±15% of pre, corresponding to decrements of approximately -37±13%, -38±12%, and -42±15%, respectively. | Peripheral fatigue depended on the measurement used; single.stimulus evoked peak force was similar across trials, whereas PS100 decreased more after the 40-min than after 3-min. |
| Junior et al., 2026 [51] | Muscle group/ size | 11 untrained males; intermittent constant-load isometric exercise in the severe-intensity domain at 115% of critical torque until task failure. | Knee extensors (KE) vs handgrip (HG) | Resting twitch decreased, -63.8±20.9% in KE vs -29±14.5% in HG; $p < 0.001$ | KE developed sibstantially greater peripheral and centrla fatigue than HG, despite a similar decline in maximal voluntary isometric contraction. |
| Dodd & Brownstein, 2026 [52] | Active muscle mass | 11 older adults (2 women); incremental recumbent cycling to task failure | Single-leg cycling (SLC) vs double-leg cycling (DLC) | Reduction in quadriceps potentiated twitch force (Qtw, pot): -45±18.8% after SLC vs -30±14% after DLC, $p = 0.049$ | Task-failure time did not differ between conditions, but conreactile impairmentwas greater after SLC. |

**Abbreviations:** ARM-CYC, cycling performed after prior high-intensity arm-cranking exercise; CYC, cycling performed without prior upper-body exercise; DLC, double-leg cycling; HG, handgrip; KE, knee extensors; MVIC, maximal voluntary isometric contraction; PS100, paired electrical stimuli at 100 Hz; Qtw,pot, potentiated quadriceps twitch force; S+, high severe-intensity trial; S−, lower severe-intensity trial; SLC, single-leg cycling.
