# Supplementary material for: Is the End-Exercise Potentiated-Twitch Decrement a Regulated Variable? A Mechanism-Oriented Review of Muscle Fatigue During High-Intensity Aerobic Exercise
Source: Muscles. 2026 Oct 9;5(4):74. doi: 10.3390/muscles5040074 (PMC13692533; doi:10.3390/muscles5040074)
Supplement: Supplementary file 1 [file muscles-05-00074-s001.zip › Table S2.pdf]

S2. Search strings adapted to PubMed/MEDLINE, Scopus, and Web of Science and records returned by each database.

Database	Search strategy	Search date
PubMed/MEDLINE	("muscle fatigue"[MeSH] OR fatigue[tiab] OR fatigability[tiab] OR "task failure"[tiab] OR "time to exhaustion"[tiab]) AND ("physical endurance"[MeSH] OR exercise[MeSH] OR endurance[tiab] OR "high-intensity"[tiab] OR "time trial"[tiab] OR cycling[tiab] OR running[tiab]) AND ("afferent pathways"[MeSH] OR "group III"[tiab] OR "group IV"[tiab] OR "voluntary activation"[tiab] OR "central fatigue"[tiab] OR twitch[tiab] OR "sarco-plasmic reticulum"[MeSH] OR "calcium release"[tiab] OR "ryanodine receptor"[tiab] OR glycogen[MeSH] OR "reactive oxygen species"[MeSH] OR potassium[MeSH] OR phos-phates[MeSH] OR "hydrogen-ion concentration"[MeSH] OR "oxygen consumption"[MeSH] OR "respiratory muscles"[MeSH] OR "perceived exertion"[tiab])	12 August 2026
Scopus	("muscle fatigue" OR fatigue OR fatigability OR "task failure" OR "time to exhaustion") AND ("physical endurance" OR exercise OR endurance OR high-intensity OR "time trial" OR cycling OR running) AND ("afferent pathways" OR "group III" OR "group IV" OR "voluntary activation" OR "central fatigue" OR twitch OR "sarco-plasmic reticulum" OR "calcium release" OR "ryanodine receptor" OR glycogen OR "reactive oxygen species" OR potassium OR phos-phates OR "hydrogen-ion concentration" OR "oxygen consump-tion" OR "respiratory muscles" OR "perceived exertion")	12 August 2026
Web of Science	("muscle fatigue" OR fatigue OR fatigability OR "task failure" OR "time to exhaustion") AND ("physical endurance" OR exercise OR endurance OR high-intensity OR "time trial" OR cycling OR running) AND ("afferent pathways" OR "group III" OR "group IV" OR "voluntary activation" OR "central fatigue" OR twitch OR "sarco-plasmic reticulum" OR "calcium release" OR "ryanodine receptor" OR glycogen OR "reactive oxygen species" OR potassium OR phos-phates OR "hydrogen-ion concentration" OR "oxygen consump-tion" OR "respiratory muscles" OR "perceived exertion")	12 August 2026
