# Supplementary material for: Is the End-Exercise Potentiated-Twitch Decrement a Regulated Variable? A Mechanism-Oriented Review of Muscle Fatigue During High-Intensity Aerobic Exercise
Source: Muscles. 2026 Oct 9;5(4):74. doi: 10.3390/muscles5040074 (PMC13692533; doi:10.3390/muscles5040074)
Supplement: Supplementary file 1 [file muscles-05-00074-s001.zip › Table S3.pdf]

Table S3. Records identified, screened, assessed, excluded with reasons, and included

**Selection stage**	**Description**	**No. of records/reports**
Records identified	Records retrieved from PubMed/MEDLINE, Scopus, and Web of Science.	1,999
Duplicates removed	Duplicate records removed before title/abstract screening.	210
Records identified	Records subjected to title/abstract screening.	1,779
Records excluded at title/abstract screening	Records classified as “Exclude” in the screening export.	283
Records retained after title/abstract screening	Records classified as “Include” and retained for subsequent assessment.	1,506
Full-text reports assessed for eligibility	Full-text reports assessed against the tiered eligibility criteria.	1,506
Full-text reports excluded	Reports not retained for the final mechanism analysis.	1,451
Reports included in the analysis	Original reports retained as evidence for the candidate mechanisms and the analyses developed in the review.	55
