# Supplementary material for: Is the End-Exercise Potentiated-Twitch Decrement a Regulated Variable? A Mechanism-Oriented Review of Muscle Fatigue During High-Intensity Aerobic Exercise
Source: Muscles. 2026 Oct 9;5(4):74. doi: 10.3390/muscles5040074 (PMC13692533; doi:10.3390/muscles5040074)
Supplement: Supplementary file 1 [file muscles-05-00074-s001.zip › Table S4.pdf]

Table S4. Evidence tier, species or experimental preparations, and training status of reports included in the analysis.

Study	Evidence tier	Species/ preparation	Population/ sample	Training status	Role in the review
Thomas et al., 2015 [4]	Tier 1	Human	13 male cyclists	Well-trained	Direct human evidence comparing peripheral and central fatigue after 4-, 20-, and 40-km cycling time trials.
Temesi et al., 2021 [5]	Tier 1	Human	32 male Trail and ultra-trail runners	Experienced trail runners	Human evidence examining the effect of race distance on neuromuscular fatigability during rail running.
Rossman et al., 2014 [6]	Tier 1	Human	8 healthy males	Recreationally active	Direct comparison of peripheral fatigue during single- vs double-leg knee-extension exercise.
Johnson et al., 2015 [7]	Tier 1	Human	8 healthy males	Moderately trained	Test the effect of prior upper-body exercise on subsequent cycling tolerance and end-exercise peripheral fatigue.
Thomas et al., 2016 [44]	Tier 1	Human	12 male cyclists	Well-trained	Compares central and peripheral fatigue across constant-load cycling intensities and durations.
Froyd et al., 2016 [50]	Tier 1	Human	12 participants (11 men, 1 woman)	Trained in endurance (>7 training sessions/week)	Examines peripheral and central fatigue during self-paced knee-extension trials of different duration.
Junior et al., 2026 [51]	Tier 1	Human	11 males	Untrained	Compares neuromuscular fatigue between knee extensors and handgrip during severe-intensity exercise.
Dodd & Brownstein, 2026 [52]	Tier 1	Human	11 older adults (2 women)	Not explicit characterized as a training category	Compares neuromuscular fatigability during incremental single- vs double-leg cycling in older adults.
Lewis et al., 2025 [1]	Tier 2	Rat skeletal muscle	Rat experimental model	N/A	Mechanistic evidence relating inorganic phosphate/ acidosis to skeletal-muscle fatigue.
Light et al., 2008 [2]	Tier 2	Dorsal root ganglion neurons innervating skeletal muscle	Experimental neuronal preparation	N/A	Mechanistic evidence that muscle afferent neurons respond to combination of protons, ATP and lactate.
Stout et al., 2026 [53]	Tier 3	Review/ conceptual framework	N/A	N/A	Conceptual positioning of Pi-afferent-effort framework.

Abbreviations: N/A, not applicable. Note: Tier 1 includes original controlled studies in healthy adults; Tier 2 includes mechanistic experiments in human fibers, rodent muscles or fibers, and isolated preparations; Tier 3 includes reviews and conceptual framework retained for analytical positioning. Training status is reported as characterized in each original source and should not be considered directly compared across studies.
