# Supplementary material for: Is the End-Exercise Potentiated-Twitch Decrement a Regulated Variable? A Mechanism-Oriented Review of Muscle Fatigue During High-Intensity Aerobic Exercise
Source: Muscles. 2026 Oct 9;5(4):74. doi: 10.3390/muscles5040074 (PMC13692533; doi:10.3390/muscles5040074)
Supplement: Supplementary file 1 [file muscles-05-00074-s001.zip › Table S5.pdf]

Table S5. Critical appraisal of the principal Tier 1 studies (sample size, design, training status, method and timing of neuromuscular assessment, main limitations)

Study	Sample size	Design	Training status	Neuromuscular assessment	Timing of assessment	Main methodological limitations	Proposition tested
Thomas et al., 2015 [4]	n = 13 men	Randomized self-paced cycling time trials of 4, 20, and 40 km.	Well-trained cyclists	MVC, VA, and potentiated quadriceps twitch (Qtw,pot) using femoral nerve stimulation.	Post-exercise assessment initiated within ~2.5 min	Small sample; recovery during the delay before post-exercise assessment may underestimate fatigue; no formal equivalence testing	Whether end-exercise peripheral fatigue converges to a common value across cycling trials of different durations
Temesi et al., 2021 [5]	n = 32 men	Observational comparison of trail races categorized as SHORT (<60 km) and LONG (>100 km).	Experienced trail runners	Knee-extensor and plantar-flexor MVC, potentiated twitch, and VA.	Post-race assessment; exact delay should be retained as reported in the original protocol	Non-randomized field design; heterogeneous race distances and environmental conditions; comparison reflects trail-running distance rather than an experimentally isolated duration effect	Whether end-exercise neuromuscular fatigability is similar across substantially different race distances
Rossman et al., 2014 [6	n = 8 men	Within-subject constant-load single- vs double-leg knee-extension exercise at 85% peak workload to exhaustion.	Recreationally active	MVC, VA, and potentiated quadriceps twitch.	~2 min after task failure	Very small sample; post-exercise delay permits partial recovery; single- vs double-leg conditions alter active muscle mass and associated systemic demand simultaneously	Whether active muscle mass influences the magnitude of peripheral fatigue tolerated at task failure
Johnson et al., 2015 [7]	n = 8 men	Cycling at 85% Wpeak with or without prior high-intensity arm exercise; isotime control included.	Moderately trained	MVC, VA, and potentiated quadriceps twitch.	First post-exercise MVC at ~2 min	Very small sample; recovery during the post-exercise delay; prior arm exercise changes systemic/perceptual state in addition to peripheral fatigue	Whether prior upper-body exercise alters cycling tolerance and the peripheral-fatigue magnitude reached at task failure
Thomas et al., 2016 [44]	n = 12 men	Randomized crossover constant-load cycling to tolerance at three intensities.	Well-trained cyclists	MVC, VA, and Qtw,pot via femoral nerve stimulation.	Within ~2.5 min after exercise	Small sample; post-exercise recovery may influence measured twitch decrement; intensity and exercise duration covary	Whether a common magnitude of peripheral fatigue is reached across different severe-intensity exercise durations/intensities
Froyd et al., 2016 [50]	n = 12 (11 men, 1 woman)	Randomized self-paced 3-, 10-, and 40-min knee-extension time trials.	Endurance- and strength-trained	MVC and electrical stimulation; SS and PS100 used to characterize peripheral fatigue.	MVC/electrical stimulation within <2 s; PS100 within <5 s	Small sample; conclusions depend partly on the peripheral-fatigue index used (SS vs PS100); single-joint model limits generalization to whole-body exercise	Whether peripheral fatigue reaches a common end-exercise value across exercise durations when measured almost immediately after cessation
Junior et al., 2026 [51]	n = 11 men	Within-subject intermittent isometric severe-intensity exercise of knee extensors and handgrip at 115% CT to task failure.	Untrained	Twitch interpolation; resting twitch for peripheral fatigue and VA for central fatigue.	Assessed at task beginning, every minute, and at task failure	Small sample; different muscle groups differ in size, architecture, fiber type, perfusion, and recruitment; no direct measures of these characteristics; caffeine intake not adequately controlled	Whether muscle group/size influences the peripheral-fatigue magnitude reached at task failure
Dodd & Brownstein, 2026 [52]	n = 11 (2 women)	Randomized incremental single- vs double-leg recumbent cycling to task failure.	Not explicitly characterized	MVC, VA, and Qtw,pot using femoral nerve stimulation.	Rapid assessment at task failure, <15 s	Small older-adult sample; incremental rather than constant-load exercise; findings represent an older population and may not generalize to trained younger athletes	Whether reducing active muscle mass permits a greater magnitude of contractile impairment at task failure
Amann et al., 2011 [32]	n = 7 men	Double-blind, placebo-controlled, randomized crossover; constant-load cycling to exhaustion al 80% Wpeak with intrathecal fentanyl vs placebo.	Male cyclists; VO2max 54.7±2.8 mL·kg^{-1}·min^{-1}	Quadriceps potentiated twitch force (Qwt, pot) via supramaximal magnetic femoral nerve stimulation; MVC, voluntary activation and EMG also assessed.	3 minutes exercise	Very small sample; post-exercise delay allows partial recovery of twitch force; fentanyl blockade also altered ventilatory and circulatory responses, so the intervention affected both afferent feedback and O_2 transport.	The group III/IV muscle afferent feedback constrains central motor drive and limits the amount/rate of peripheral fatigue accumulated during high-intensity endurance exercise.
Amman et al., 2009 [31]	n = 8 men	Double-blind, placebo-controlled, randomized crossover; three 5-km cycling time trials:	Competitive male cyclists	Quadriceps potentiated twitch force (Qwt, pot) via supramaximal magnetic femoral nerve stimulation; MVC, voluntary activation, M-wave	3 min after exercise	Very small sample, 3-min post-exercise delay allows partial recovery of twitch force; intrathecal fentanyl altered afferent feedback and was accompanied by changes in ventilation, arterial O_2 saturation and blood	The attenuating group III/IV muscle afferent feedback increases central motor drive and permits peripheral fatigue to exceed the reproducible end-

Table S5. Critical appraisal of the principal Tier 1 studies (sample size, design, training status, method and timing of neuromuscular assessment, main limitations)

		control, placebo, and intrathecal fentanyl.		characteristics and vastus lateralis iEMG.		lactate, so the intervention was not physiologically isolated to motor-control effects alone.	exercise level normally reached with intact feedback.
Amman & Dempsey, 2008 [48]	n = 8 men	Interventional crossover study. Participants first completed two constant-workload prefatigued trials producing different levels of quadriceps fatigue, then on separate randomized days completed three 5-km cycling time trails: control, moderate prefatigued, and severe prefatigued.	Competitive male cyclists	Quadriceps potentiated twitch force (Qwt, pot) via supramaximal magnetic femoral nerve stimulation; MVC, voluntary activation, M-wave characteristics and quadriceps EMG/iEMG.	4 min after exercise	Very small sample; 4-min post-exercise delay allows substantial recovery of twitch force; prefatigued manipulation changes several physiological variables simultaneously; central motor drive was inferred from surface EMG, which the authors acknowledge cannot quantify the exact contribution of central neural drive to power output.	Differing levels of pre-existing locomotor muscle fatigue alter central motor drive and exercise performance while end-exercise peripheral fatigue converges toward a similar task-specific value.
Blain et al., 2016 [15]	n = 8 men	Counterbalanced crossover; 5-km cycling time trial under control vs lumbar intrathecal fentanyl, with vastus lateralis muscle biopsies.	Healthy, recreationally active males.	Potentiated quadriceps twitch torque using electrical femoral nerve stimulation: single twitch (QTSingle), paired 10-Hz and 100-Hz stimulation; MVC voluntary activation, M-wave and EMG also assessed.	1 min after exercise, repeated al 1, 2, 4, 6, 15 min, 5 h and 24 h.	Small sample; 1-min delay still permits some recovery; fentanyl blockade also altered ventilatory/Cardiovascular responses and therefore muscle O2 delivery; exact degree of afferent blockade and relative contribution of mechano- vs metabosensitive fibers were unknown; surface EMG provides only an indirect estimate of motoneural output.	The attenuating group III/IV muscle afferent feedback permits greater intramuscular metabolic perturbation and peripheral fatigue during whole-body endurance exercise.
Bangsbo et al., 1996 [16]	n = 7 men	Randomized within-subject comparison of exhaustive single-leg knee extension exercise performed with (HL) or without (control) prior intense, intermittent arm exercise.	Healthy, usually physically active male university students	No direct evoked neuromuscular assessment. Fatigue was operationalized as failure to maintain the required power output; muscle biopsies, leg blood flow and arterial/venous blood sampling were used.	Vastus lateralis biopsies obtained before and immediately after exhaustive leg exercise.	No direct twitch/VA measure of peripheral or central fatigue; the metabolic manipulation was produced through prior arm exercise and therefore was not an isolated manipulation of muscle pH.	Elevated muscle acidity alters muscle metabolism and contributes to fatigue during intense exercise.
Amann et al., 2006 [36]	n = 8 men	Randomized repeated-measures study involving four 5-km cycling time trials across different arterial O_2 contents induced by FIO_2 manipulation; additional constant-work-rate exercise was also used	Healthy, trained male cyclists	Supramaximal magnetic femoral nerve stimulation (Qtw, 1–100 Hz); quadriceps EMG used to estimate central neural drive	Neuromuscular function assessed before, 2.5 min and 35 min after exercise for the time trials	Small sample; initial neuromuscular assessment occurred 2.5 min after exercise; changing FIO_2/CaO_2 has systemic effects throughout the organism rather than acting solely on locomotor muscle	Arterial O_2 content modifies performance and central motor output through its influence on peripheral locomotor-muscle fatigue
Amann et al., 2007 [37]	n = 8 men	Randomized, blinded constant-load cycling to exhaustion under normoxia, moderate hypoxia and severe hypoxia; acute hyperoxygenation applied at task failure.	Competitive male cyclists	Potentiated quadriceps twitch force (Qtw,pot) using supramaximal femoral nerve stimulation; MVC, voluntary activation and quadriceps EMG also assessed.	Assessment performed before and 2 min after exercise.	Small sample; 2-min post-exercise delay; severe hypoxia introduces a CNS hypoxic influence in addition to effects on peripheral muscle fatigue.	The relative contribution of peripheral versus central mechanisms to exercise limitation changes as arterial hypoxemia becomes more severe.
Romer et al., 2007 [38]	n = 9 men	Randomized/balanced constant-load cycling at ~92% peak power in normoxia to exhaustion, severe hypoxia to	Competitive endurance athletes; 7 of 9 were cyclists	Supramaximal magnetic femoral nerve stimulation using non-potentiated and potentiated single twitches and paired stimuli (10–100 Hz)	Pre-exercise and 2.5, 35 and 70 min post-exercise	Small sample; first neuromuscular measurement at 2.5 min permits some recovery; severe hypoxia simultaneously changes several physiological determinants of exercise tolerance.	Whether acute severe hypoxia accelerates peripheral locomotor-muscle fatigue and whether this contributes to reduced endurance capacity.

Table S5. Critical appraisal of the principal Tier 1 studies (sample size, design, training status, method and timing of neuromuscular assessment, main limitations)

		exhaustion, and normoxic isotime control.					
Romer et al., 2006 [39]	n = 8 men	Randomized, counterbalanced repeated-measures cycling study manipulating inspiratory muscle work through proportional assist ventilation (PAV) and inspiratory resistive loading (IRL), with matched control trials.	Endurance-trained male cyclists	Supramaximal magnetic femoral nerve stimulation; quadriceps twitch responses across 1–100 Hz and assessment of membrane excitability.	Pre-exercise and 2.5, 35 and 70 min post-exercise.	Small sample; participants could not be blinded to respiratory treatments; first fatigue assessment occurred 2.5 min after exercise.	Respiratory muscle work contributes to locomotor-muscle peripheral fatigue during sustained high-intensity exercise.
Salam et al., 2018 [45]	n = 11 men	Repeated TTE cycling tests used to determine CP and W′ following either a 30-min mentally fatiguing task or control task.	Well-trained male cyclists; >5 h training/week for ≥3 years	No direct neuromuscular assessment. Outcomes included TTE, CP, W′, perception of effort, HR and blood lactate.	N/A for neuromuscular assessment.	Small male-only sample; no direct measurement of central or peripheral neuromuscular fatigue, so the physiological basis of the reduced W′ cannot be established from neuromuscular measurements.	Experimentally increasing mental fatigue/perception of effort alters critical power and W′ during cycling.
Marcora et al., 2009 [46]	n = 16 (10 men, 6 women)	Single-blind, randomized, counterbalanced crossover; cycling to exhaustion at 80% peak power after 90 min of demanding cognitive activity vs neutral documentaries.	Healthy adults engaged in regular aerobic training	No direct neuromuscular assessment. Cardiorespiratory responses, perceived exertion, motivation and related physiological measures were assessed.	N/A for neuromuscular assessment.	No direct measure of peripheral or central neuromuscular fatigue; proposed mechanism is based primarily on altered perception of effort alongside largely unchanged physiological responses.	Prior mental fatigue reduces high-intensity endurance exercise tolerance through increased perception of effort rather than cardiorespiratory or musculoenergetic alterations.

Note: This table provides a qualitative critical appraisal rather than a formal risk-of-bias assessment. Limitations are reported in relation to the interpretation of neuromuscular fatigue and the specific proposition tested by each study. Small sample sizes and differences in the timing and method oof post-exercise neuromuscular assessment should be considered when comparing studies. None of the studies testing between-condition differences formally assessed equivalence.

Abbreviations: CaO_2, arterial oxygen content; CNS, central nervous system; CP, critical power; CT, critical torque; EMG, electromyography; FIO_2, fraction of inspired oxygen; HR, heart rate; MVC, maximal voluntary contraction; PE, perception of effort; PS100, paired electrical stimulation at 100 Hz; Qtw, quadriceps twitch force; Qtw,pot, potentiated quadriceps twitch force; RPE, rating of perceived exertion; SpO_2, peripheral oxygen saturation; SS, single stimulus; TTE, time to exhaustion; VA, voluntary activation; W′, curvature constant of the power–duration relationship; Wpeak, peak power output.
